# Seroprevalence of Fecal-Oral Transmitted Hepatitis A and E Virus Antibodies in Burkina Faso

**DOI:** 10.1371/journal.pone.0048125

**Published:** 2012-10-22

**Authors:** Kuan Abdoulaye Traoré, Hortense Rouamba, Yacouba Nébié, Mahamadou Sanou, Alfred S. Traoré, Nicolas Barro, Pierre Roques

**Affiliations:** 1 Centre de Recherche en Sciences Biologique Alimentaire Nutritionnelles (CRSBAN), Université de Ouagadougou, Ouagadougou, Burkina Faso; 2 Centre National de Transfusion Sanguine, Ouagadougou, Burkina Faso; 3 Centre médical de Samandin, Ouagadougou, Burkina Faso; 4 Division of Immuno-Virologie, Institute of Emerging Diseases and Innovative Therapies, Commissariat à l'Energie Atomique (CEA), Fontenay-aux-Roses, France; 5 Unite Mixte de Recherche E1, University Paris Sud 11, Orsay, France; Agency for Science, Technology and Research – Singapore Immunology Network, Singapore

## Abstract

Hepatitis A virus (HAV) and hepatitis E virus (HEV) infections occur chiefly as a result of unhygienic conditions. The purpose of this study was to assess the seroprevalence of antibodies to both viruses in central Burkina Faso in the absence of a recorded hepatitis epidemic. Serum samples from 178 blood donors (131 males and 47 females) and from 189 pregnant women were collected from November 2010 to March 2012, at blood banks and medical centers in Burkina Faso. An immunochromatography test was used to screen for Anti-HAV IgM and IgG in a subgroup of 91 blood donors and 100 pregnant women. The seroprevalence of anti-HAV IgG was 14.3% [CI95, 7.1–21.4%] for all blood donors and 23% [CI95, 14.8–31.2%] for pregnant women. Anti-HEV IgG were detected using the ELISA kits Dia.pro and Wantai and were found in 19.1% [CI95, 13.3–24.9%] of the blood donors and 11.6% [CI95, 7.1–16.2%] of the pregnant women. The seroprevalences of anti-HAV and anti-HEV IgGs did not differ significantly between men and women blood donors. Anti-HAV IgM was detected in 3.3% of the blood donors and in 2% of the pregnant women. These findings for asymptomatic individuals indicate that the HAV and HEV circulate at low but significant levels. This is the first evaluation of the acute hepatitis virus burden in Burkina Faso and the underlying epidemiologic status of the population.

## Introduction

Hepatitis A virus (HAV) and hepatitis E virus (HEV) are the leading causes of acute viral hepatitis in the world. In developing countries, these infections are associated with poor hygiene and, in particular, the lack of clean drinking water and, in some areas, inadequate sanitation [1]–[3]. It is estimated that 1.5 million new HAV infections occur each year and that one-third of the world's population is infected with HEV [4]. These viruses spread by the fecal-oral route usually through contact between people [5] or by ingestion of contaminated food or water [6].

HAV belongs to the genus *Hepatovirus* and is a member of the *Picornaviridae* family [7]–[9]. HAV is a non-enveloped virus 27–32 nm in diameter. The viral genome is a single-stranded RNA of positive polarity, which is non-segmented and is packaged in an icosahedral capsid. HAV strains belong to a single serotype. However, there are seven viral genotypes (I–VII) and viral strains of the same genotype share greater than 85% nucleotide identity. Viruses of four of the genotypes (I, II, III and VII) have been recovered from human HAV cases, whereas viruses of the other three genotypes (IV, V and VI) have each been isolated only from a different simian species developing a hepatitis A-like illness during captivity [10], [11]. HAV occurs in all countries of the world and the incidence of infection varies with the local socioeconomic conditions and health standards [9]. HAV infection is almost always clinically silent in children under three years of age but is usually symptomatic in adults and is sometimes severe. In infected individuals aged 40 years or more, the mortality rate exceeds 2% [12].

HEV was classified as a new *Hepeviridae* family on the basis of its structure and genome organization. The *Hepevirus* genus is still the only known member of this family [13]. HEV is a non-enveloped cubic-shaped virus 27–33 nm in diameter and has tooth-like projections on its surface. The viral genome consists of a single-stranded RNA of positive polarity. Four major HEV genotypes, subdivided into 24 subtypes (some being the result of recombination), have been identified through classification and regional genotypic analyses [14], The different genotypes exhibit 72–77% sequence identity and there is 85–90% identity within subtypes [14], [15]. Unlike other hepatitis viruses, humans are not the only natural host of this virus. Recent work shows that there are large reservoirs of HEV in various animal species, especially pigs, rabbits and ferrets, suggesting that HEV is a zoonotic agent [16], [17]. HEV has an extensive global distribution, is endemic in many poor countries and causes occasional epidemics [13]. In developed countries, many of the HEV infections are imported, although there is low background of local infection [18], [19]. The endemic background in Western Europe is higher than in other developed zones, and is mainly associated with genotype 3 viruses and a potential reservoir in pigs [20]–[23]. Although the symptoms are generally mild, HEV infection is responsible for high mortality rates (up to 20%) in pregnant women, especially during the three first month of pregnancy [24]–[26]. HEV is endemic in regions where the water supply may be contaminated with animal waste, such as in Central Asia, the Middle East, and parts of South America and Africa. There have been major waterborne epidemics in refugee camps in Somalia [27] and Sudan [28], causing sickness and death. The groups of people at risk include those with liver disease, the elderly and the immunocompromised [29], [30].

Thus, HAV and HEV are both food and waterborne pathogens. They share the same routes and mechanisms of spread and they both cause similarly severe epidemics.

Although we conducted preliminary evaluations of food-borne viruses In Burkina Faso in 2005 and 2008, little is known about the prevalence of HAV and HEV in food [31], [32]. The inhabitants of Ouagadougou are undoubtedly exposed to these viruses as a consequence of the socioeconomic situation, the hygiene standards and the nutritional habits marked by the consumption of street-vended products [32]. Indeed, an evaluation of the etiology of severe diarrhea in infants in Burkina Faso demonstrated the involvement of rotavirus and adenovirus in these disease outbreaks. Both of these viruses are, like HEV and HAV, transmitted by the fecal-oral route [33], [34]. Here, we report an analysis of the seroprevalence of HAV- and HEV-specific antibodies in the population of Burkina Faso.

## Materials and Methods

### Ethics statement

This study was carried out according to the routine practices of the Regional Blood Transfusion Center of Ouagadougou (RBTC-O) and the Medical Center Surgical unit of Samadin (MCS), and was approved by the Ethics Committee for Health Research (ECHR): “Comité national d'éthique pour la recherche en santé (CNERS). Ministère de la Santé 03 BP 7009. Ouagadougou 03, Burkina Faso”. All subjects participating in the study signed a voluntary consent form after being given all the information necessary and sufficient to make an informed decision regarding their participation in this study.

### Study design and population

The study was carried out between November 2010 and January 2011, and then in March 2012. The study population of blood donors and pregnant women was classified into representative groups. Serum samples from 178 blood donors and 189 pregnant women attending the Medical Center of Samadin for prenatal HIV testing who were found to be fit to donate blood after the medical interview were used (367 samples in total). Blood samples were collected according to the international rules of blood donation as described by Rouger (2006) [35].

### Sampling

A volume of 5 ml of whole blood from each donor was obtained by venipuncture and collected into a dry tube. Blood samples were transported to the laboratory and kept at 4°C for overnight for sedimentation. The samples were then centrifuged at 3000 *g* for 10 min at 25°C. Sera were gently collected into cryotubes (Nalgene®) and stored at −20±5°C until the serological analysis.

### Serological tests

All serum samples were analyzed for specific anti-HAV IgM and IgG using an immunochromatographic test (SD Bioline IgM/IgG anti-HAV; Standard Diagnostics, Inc., Korea) according to the manufacturer's instructions. This test has a sensitivity of 97.6% and a specificity of 98.0%. Serum samples were also screened for anti-HEV IgG by enzyme linked immunosorbent assay (ELISA) tests (DIA.PRO Diagnostic Bioprobe Srt, Italy or Beijing Wantai Biological Pharmacy Enterprise CO., LTD, China). Positive and negative controls were included in all the ELISA microplates. The results were scored as positive or negative according to the standard procedures recommended by the manufacturer.

### Data processing

The data were processed, analyzed and plotted using Excel 2007 and GraphPad Prism 5.0d. 95% confidence intervals were calculated (CI95); Chi-2 and corrected P values were obtained using StatView 5.0 software (SAS Institute, USA).

## Results

### Seroprevalence of anti-HAV immunoglobulin

The results of screening blood donors and pregnant women for anti-HAV IgM and IgG are shown in Table 1. Nine samples from blood donors did not give results that could be interpreted.

**Table 1:** Prevalence of anti-HAV IgG and IgM in pregnant women and blood donors.

Serological markers	Blood donors		Pregnant women (n = 100)
	Women (n = 22)	Men (n = 69)	
IgG	4 (18.2%, CI95 [2–34%])	9 (13%, CI95 [5–20%])	23 (23% CI95 [14–31%])
IgM	1 (4.5%)	2 (3%)	2 (2%)
IgG + IgM	1 (4.5%)	2 (3%)	2 (2%)

The donor population included more men 69/91 than women 22/91, but the prevalence of anti-HAV IgG did not differ between women (18%, CI95 [2–34%]) and men (13%, CI95 [5–20%]). The seroprevalence of anti-HEV was slightly higher among pregnant women (23%, CI95 [14–31%]) than blood donors. The prevalence of anti-HAV IgM was similar for all three groups. The presence of anti-HAV IgM indicates a history of recent HAV infection, but as blood donors are questioned about clinical illness before blood is collected, any these infections were presumably asymptomatic.

**Figure 1:**
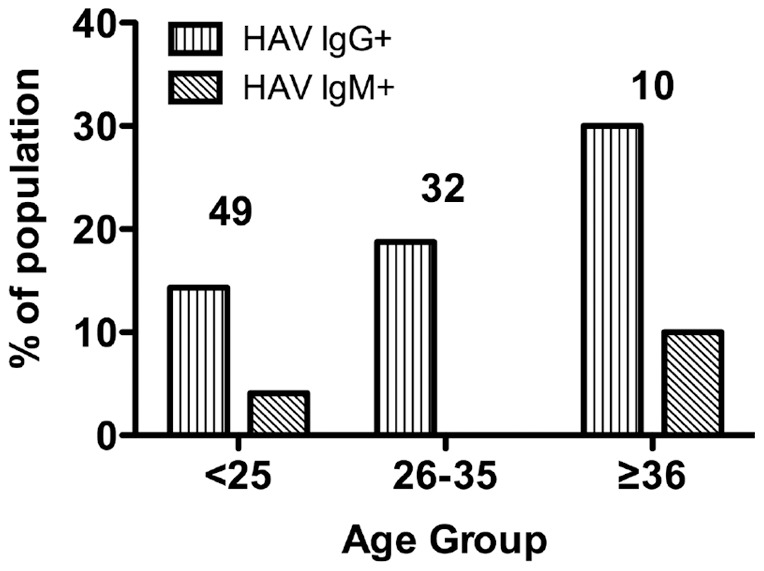
Distribution of anti-HAV IgG and IgM in blood donors according to age group.

The prevalence of anti-HAV antibodies according to age is shown in Figure 1. The anti-HAV prevalence increased slightly from the <25 age group to the >36 age group. Anti-HAV seroprevalence was high in the 46–55 years age group, whereas no positive cases were detected in the 56–65 years age group (data not shown). There were no significant differences between the age groups and no trend for a decrease or an increase of prevalence with age. Few subjects were anti-HAV IgM positive, and all positive subjects were <25 or 36–45 years old.

### Seroprevalence of anti-HEV immunoglobulin

**Figure 2:**
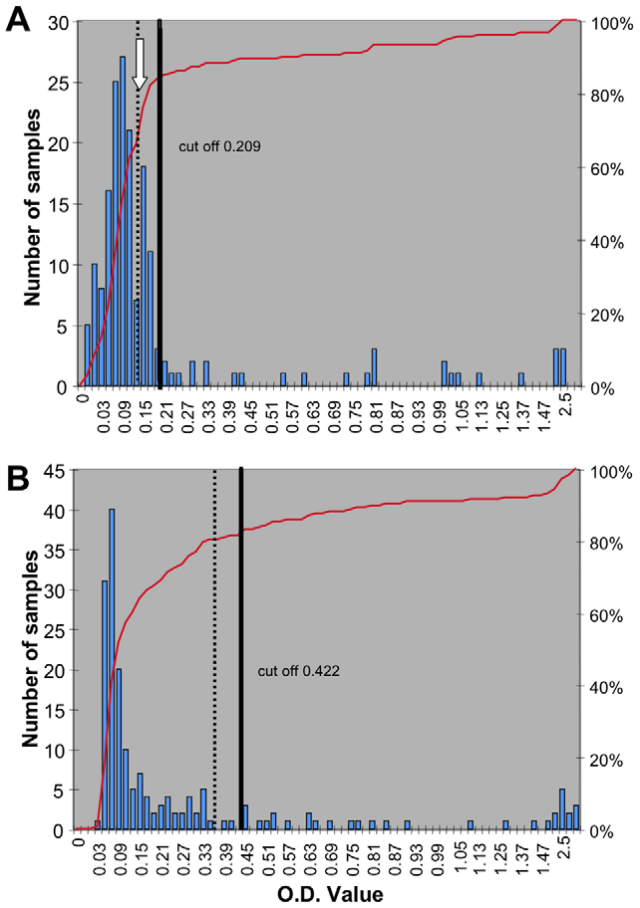
Comparison of results of testing with IgG ELISA kits. A) Wantai kit B) Dia.Pro test. Number of samples per OD value class (blue bars); red line: cumulative % of value: 100% is the 180 tested samples. Negative control value is given by a vertical doted line and the cut-off value as determined for the kit is given by a vertical continuous line. Arrows in the Wantai graph (A) indicate the OD values for a group of people with sub-detectable IgG.

**Figure 3:**
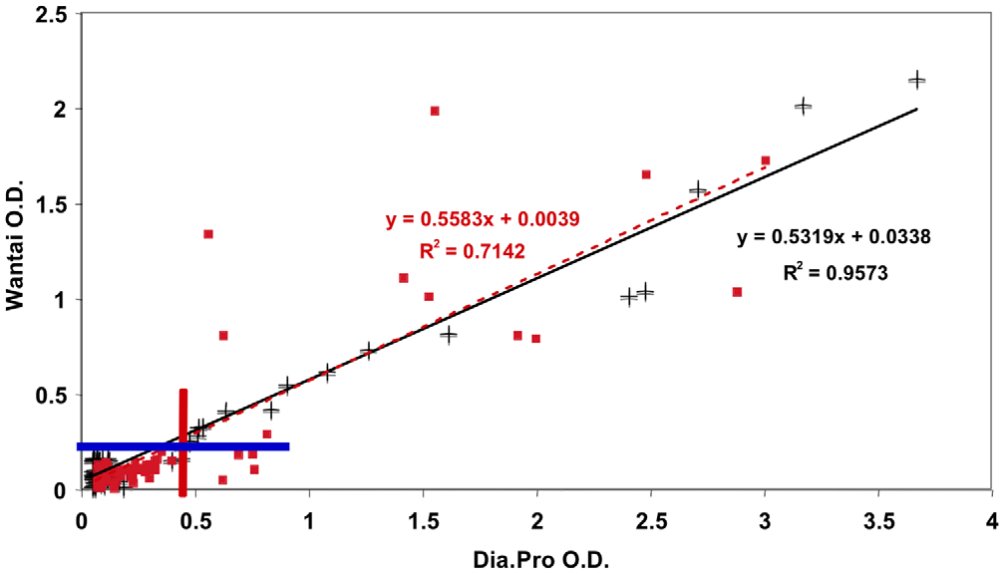
Evaluation of relation-ship between Wantai and Dia.Pro test results in positive samples. Crosses indicate blood donor samples and red squares samples from pregnant women. Linear correlations are significant within the two group but with significantly less dispersion of the blood donor samples than of the samples from pregnant women. The blue line indicates the cut-off level for the Wantai kit, and the red line the cut-off for the Dia.Pro kit.

Pregnant women and in blood donors were screened for anti-HEV IgG (table 2 shows the prevalence according to age and group). There was no significant difference between general prevalence in January 2011 and that found by subsequent analysis in March 2012. There is recent evidence from England and France that a new ELISA test (the Wantai kit) detects previously unrecognized cases of anti-HEV seropositivity. We therefore compared the values obtained with the Dia.Pro test and the newly available Wantai kit for the samples collected from the blood donor group in 2011. With the Dia.Pro test, 19.1% of 89 blood donor samples from 2011 were positive, whereas 14.6% of the same samples scored positive and 4.5% indeterminate in the Wantai test. We repeated the comparison in a new set of sera obtained in 2012 from blood donors and pregnant women. The distribution of optical density (O.D.) values obtained for the 189 samples with the Dia.Pro and Wantai tests are shown in Figure 2. Despite a large difference in the range of positive values between the two kits, the total number of positive samples was not very different; however, as for the previous comparison, samples scored as indeterminate in the Wantai test were positive in the Dia.Pro test (five sera). There was a very good correlation between the O.D. values for the two tests (Figure 3). Although the slopes were the same for the two groups, the correlation coefficient was higher for blood donors (r^2^ = 0.95) than for pregnant women (r^2^ = 0.71); indeed, the correlation for pregnant women showed a significant deviation from the linearity (Runs test p = 0.0092).

**Table 2:** Prevalence of anti-HEV IgG in pregnant women and blood donors per sampling period (Dia.Pro test).

Year and marker	Blood donors		Pregnant women
2011	Women (n = 22)	Men (n = 67)	(n = 100)
IgG	5 (23% CI95 [5–40%])	11 (16.4% CI95 [7–25%])	9 (9% CI95 [3–14%])
March 2012	Women (n = 25)	Men (n = 64)	(n = 89)
IgG	6 (24% CI95 [7–40%])	12 (18.7% CI95 [9–28%])	13 (9% CI95 [7–22%])

Dia.Pro test data were used for all subsequent analyses of prevalence (Table 2). The anti-HEV IgG seroprevalence in female blood donors (11/47, 23%, CI95 [7–40%]) was not significantly different from that in male donors (23/131, 16%, CI95 [11–24%]). The prevalence of anti-HEV IgG among pregnant women (11%, CI95 [7–16%]) was lower than that in the entire blood donor group (19% CI95 [13–25%]); the difference with the female blood donor (non pregnant) group was highly significant, with an Odds Ratio ratio of 2.3 (CI95 [1.7–3.24], χ^2^ =  4.31, p<0.05). This may have been because the women blood donors were older than the pregnant women.

**Figure 4:**
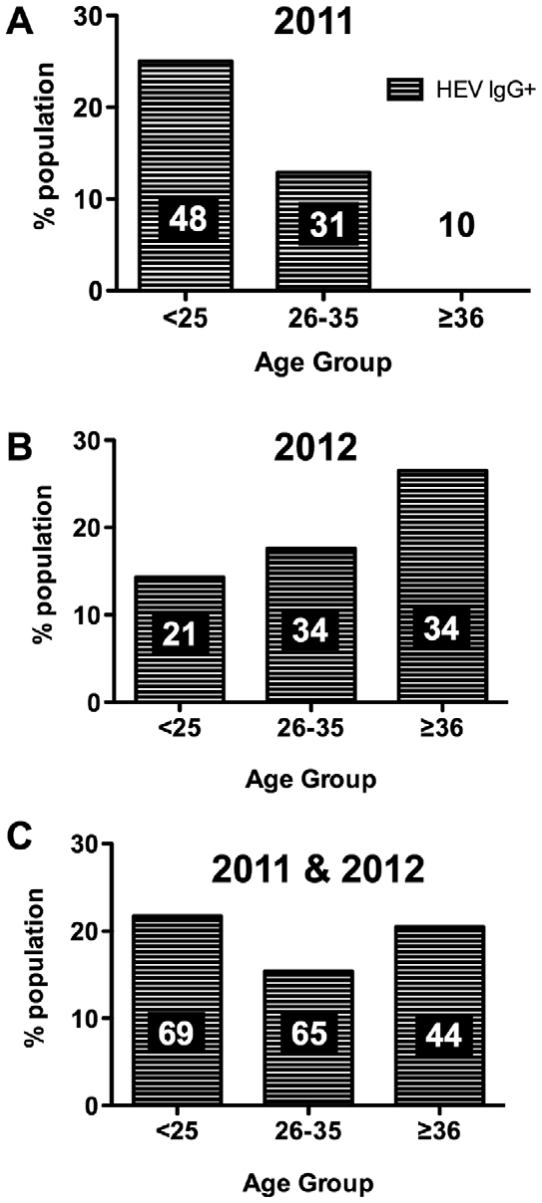
Distribution of anti-HEV IgG in blood donors according to age group. A) in January 2011; B) in March 2012; C) groups 2011 and 2012 combined.

This difference between pregnant women and blood donors remained significant when the data from the two sampling dates were analyzed independently. However, there was an unexpected large difference between the distributions of seroprevalence according to age in 2011 and 2012 (Figure 4A, 4B). In 2011 anti-HEV IgG seropositive blood donors were all in the <25 and 26–35 age groups, and there were no seropositive in the older age groups; the decrease in seroprevalence with age was significant (χ^2^ =  4.4 p = 0.038 (Figure 4A). This unexpected pattern was not found with the samples collected in 2012 which, by contrast, showed an increase of seroprevalence with age (but not significant χ^2^ =  1.32 p = 0.257; Figure 4B).

## Discussion

This study investigating the prevalence of anti-HAV and anti-HEV seropositivity in Ouagadougou is the first of its kind in Burkina Faso. For epidemiological purposes, blood donors can be considered to approximate to the healthy “general population”. However, the age criteria for blood donation and the elimination of samples found to be positive for hepatitis B and C antigens or for HIV antibodies could lead to an underestimation of the prevalence of anti-HAV and anti-HEV seropositivity in the general population. The group of pregnant women was not subject to such limitations but may have presented other biases as it included only young adult women attending prenatal clinics. Nevertheless, these two groups together may be fairly representative of the general population.

Hepatitis, manifest as jaundice, is common in Burkina Faso, but biological investigations are often conducted late, limited to chronic disease and involve only HCV and HBV testing. Cases of acute hepatitis may be diagnosed without determining either the virology of the infection or whether the jaundice is HBV- (HbsAg) negative. There have been few investigations of anti-HAV seroprevalence [36]. Our study detected HAV infection endemicities of 14.3% (CI95 [7.1–21.5%]) in blood donors and 23% (CI95 [14–31%]) in pregnant women. The difference between these two groups could be due by the close proximity of these women to children who are considered to be potential carriers of the virus [37], [38]. The seroprevalence of anti-HAV IgM indicates that the incidence of infections is relatively high and that there is a risk of major outbreaks if vaccination is not continued.

The proportions of anti-HAV seropositives in the different age groups of blood donors revealed a high prevalence (50%) in the group of 46–55 year olds. The different rates of seropositivity between the different age groups suggests age-related HAV exposure in Burkina Faso, a phenomenon probably associated with the severity of the disease increasing with age. In the United States, the hospitalization rates for hepatitis A infections are 3% for individuals younger than 18 years and 13% for individuals older than 18 years. The fatality rate is 0.3% overall, but it is higher than 2% for individuals over 40 years old [12]. The seroprevalence we observed in the 26–35 year old group (18.7%) agreed with the findings of Dubois *et al*. (1992), Denis *et al*. (2003), and Poda (2010) [36], [39], [40], and is consistent with the idea that young adults are the most highly exposed and sensitive to infection. Indeed, the dietary habits of young adults, who frequently choose ready-to-eat food and fast-food, have been recognized as health-risk factors because poor quality control and undercooking of these products are commonplace in Burkina Faso [32]. Studies in other emerging or developing countries around the world have shown that the prevalence of HAV infection decreases as the living conditions of the populations improve [41]–[46]. This in accordance with the situation in Burkina Faso characterized by its low income and the poor hygienic status of a major part of population in the capital Ouagadougou, associated with high prevalence of hepatitis. Indeed, the causes of acute hepatitis, the most common clinical sign of HAV and HEV infection, led us to evaluate the prevalence of these two viruses in parallel in our population.

Recently, the use of the “Wantai” kit in the UK and France led to the identification of higher rates of HEV positivity than suggested by previous studies using the Dia.pro kit. However, when we tested our Burkina Faso cohort in blind using both methods we found a very similar result for the two tests. If this result was related to the genotype of the circulating HEV or to a specific immune status in the Burkinabe population remained to be assessed. The seroprevalence of anti-HEV IgG among Ouagadougou blood donors (19.1%, CI95 [13–24%]) was higher than those reported for Italy (2.6%), Germany (2.1%) and California (1.2%) but was close to that found in the south of France (16.6%) [47]–[49]. The seroprevalence of HEV found in this study was similar to those observed in Tunisia (22%), Burundi (14%), Singapore (14.5%) and South Africa (15.3%) [50]–[52]. The anti-HEV seroprevalence in our group of pregnant women (11.6%, CI95 [7.1–16.2%]) from the Burkina Faso plateau region of which Ouagadougou is the center, was lower than those found in most of these other countries but was comparable to those found in pregnant women in Tunisia (12.1%) and Turkey (12.6%) [53], [54]. Low prevalences (1–5%) have been reported for countries with low HEV endemicity, such as the United States or those in Northern Europe [55], [13]. The HEV seroprevalence among pregnant women (11.6%) was slightly lower than that of either men or women blood donors (17.6% and 23.4%, respectively). Other studies of pregnant women reported similar patterns in Gabon (10–20%). These findings may be related to differences in socioeconomic status of the pregnant women, but we did not record any such data in our study [56]. We found the highest anti-HEV seroprevalence in the <25 years of age group (Figure 4C), which has been reported to be at high risk for HEV infection worldwide [13], [57], [58]. Nevertheless, in endemic areas, childhood hepatitis E infections are frequent than childhood hepatitis A infections. HEV is an infection of young adults, with the peak incidence between the ages of 15 and 35 years [59], [60]. Our study was limited to this age class and we did not examine the seroprevalence in children. As HEV infection is particularly serious during pregnancy, our findings indicate that HEV awareness needs to be increased. The distribution of IgG anti-HEV seropositivity among blood donors according to age group showed a significant increase for the age group >36 years, from about 0% in 2011 to 26.4% in 2012. This absence found in the first sampling period in 2011 was quite unusual but could be due to sampling hazard or to HEV being introduced only recently. The 2012 data were more in accordance with those obtained in China by Li *et al.* in 2006 [61], showing that the seroprevalence of HEV could be related to both age and the level of endemicity or dietary exposure factors.

Recent studies in France indicate that HEV higher seroprevalence may be associated with dietary habits, and particularly with consumption of pork or rabbit meat [14], [16], [20], [49]. Despite presence of rabbit breeding in rural areas of Bukina-Faso, little is known about rabbit meat consumption in large cities. In Ouagadougou, pork from locally-bred pigs is found in local fast-foods (grilled pork vending outlets) and is widely consumed by the population. As shown recently, the nature of processing affects the prevalence of HEV in the pork production chain in Europe [62]. Thus, complementary studies addressing eating habits and the serology of exposed population would be the logical continuation of the present study. Several studies have shown that low socioeconomic status, poor hygiene, lack of sewers, and underdeveloped health care facilities constitute the main transmission risk factors for HAV and HEV [32], [63], [64].

## Conclusions

Despite being limited to a small subset of the Ouagadougou population, our findings emphasize the need to establish a national HAV and HEV surveillance program consisting. This program should include additional sero-epidemiological surveys to track changes in the epidemiology of these viruses, and especially factors for their persistence, and the role of eating habits and food processing methods in the spread of these infections. Data from such a program could be used as the basis for designing an HAV vaccination campaign and to enhance HEV surveillance.

In addition, HEV infection should not be considered rare and its presence should be actively investigated, especially in pregnant women given that HEV infection adds significant health risks to pregnancy. Additional studies are needed to detail the incidence and severity of this illness in pregnant women in Burkina Faso.

Finally, addressing the public health problems associated with the enteric transmission of viral hepatitis in developing countries will require implementing stronger measures to prevent fecal contamination of food and water.
